# Datasets of high-resolution water level and discharge from the Saigon-Dong Nai estuary system impacted by a developing megacity, Ho Chi Minh City - Vietnam

**DOI:** 10.1016/j.dib.2023.109147

**Published:** 2023-04-13

**Authors:** Francisco Rodrigues do Amaral, Tin Nguyen Trung, Thierry Pellarin, Nicolas Gratiot

**Affiliations:** a Université Grenoble Alpes, CNRS, INRAE, IRD, Grenoble INP, IGE, 38000 Grenoble, France; b CARE, Ho Chi Minh City University of Technology, VNU-HCM, Vietnam

**Keywords:** Tidal river, Low-cost installation, ADCP, Tidal propagation in rivers

## Abstract

We present a new hydrological dataset collected during a field campaign in the Saigon-Dong Nai estuary system, Vietnam. These data include water level and water temperature measurements at five locations along the Saigon river and 2 locations along the Dong Nai river as well as discharge measurements from four 24-hour Acoustic Doppler Current Profiler (ADCP) campaigns at 2 locations in the Saigon river and 1 location in the Dong Nai river. Additionally, water level was barometrically compensated using air pressure measurements. Data were sampled between October 21^st^, 2022 and December 16^th^, 2022 and are provided in three processing stages namely, direct measurements as provided by the sensors (raw), barometricaly compensated measurements (pre-processed) and corrected measurements (post-processed). Even though of short duration (about 2 months), this dataset provides water level measurements at unprecedented spatial and temporal resolution in a region where data is scarce and not freely available. The synchronous logging of multiple water level sensors along river provides an opportunity to study profiles of water surface slope and upstream tidal propagation. Furthermore, the concurrent discharge measurements can be used to calibrate hydrological and/or hydraulic models of this estuary system. Additionally, the spatial resolution of this dataset is similar to the prospective measurements that the novel Surface Water and Ocean Topography (SWOT) satellite will provide. Thus, it enables the study of synthetic SWOT measurements to evaluate the future potential of the SWOT satellite over this estuary system.

**Specifications Table**

**Table:** 

Subject:	Water Science and Technology
Specific subject area:	Water level and discharge measurements in tidal rivers using low-cost installation of water level loggers and 24-hour ADCP campaigns.
Type of data:	Table Graph
How the data were acquired:	Water level: Submersed brick-and-pipe installation of Onset HOBO U20L-01 water level loggers measuring absolute pressure and temperature. The HOBOware Pro version 3.7.21 software is used to convert absolute pressure into water level and perform barometric compensation. Air pressure: Measurements from a Watson W-8681-MKII wireless weather station are used as barometric reference. Discharge: 24-hour campaigns using the Workhorse Rio Grande Acoustic Doppler Current Profiler (ADCP) measuring discharge twice hourly.
Data format:	Raw Pre-processed Post-processed
Description of data collection:	Water level measurements were performed at 5 locations in the Saigon river branch and at 2 locations in the Dong Nai river branch between October 21^st^, 2022 and December 16^th^, 2022. We perform barometric compensation, identify and correct of out-of-water instances using temperature and pressure readings and quadratic interpolation. Discharge measurements were performed at 2 locations in the Saigon river and 1 location in the Dong Nai river. We use the free, open-source QrevInt software for post-processing and quality control and obtain mean discharge, mean width and mean river cross-section. The uncertainty in discharge is computed using the OURSIN framework [1].
Data source location:	City/Town/Region: Ho Chi Minh City Country: Vietnam Latitude and longitude (and GPS coordinates, if possible) for collected samples/data: presented in Table 1.
Data accessibility:	In a public repository. Repository name: DataSuds Data identification number: doi.org/10.23708/NKQDNB Direct URL to data: https://doi.org/10.23708/NKQDNB Full reference: Rodrigues Do Amaral, Francisco; Nguyen, Trung Tin; Gratiot, Nicolas; Pellarin, Thierry, 2023, "High-resolution water level and discharge measurements from the Saigon-Dong Nai estuary system, Vietnam, 2022", https://doi.org/10.23708/NKQDNB, DataSuds, V1, UNF:6:mWcp4LMDvHYz+oaLv15f4w== [fileUNF]

## Value of the Data

• The new water level dataset provides a series of unprecedented measurements over the Saigon-Dong Nai estuary system at high time (every 15 minutes) and space (every 10 km along river) resolution. The new discharge dataset encompasses four 24-hour ADCP measurements at three locations providing discharge measurements during different tidal regimes.
• Can benefit environmental protection and management agencies with a more reliable forecasting of river water levels and better decision making by researchers and policy makers regarding urban flooding and dam management.
• Can be used to verify and validate remote sensing measurements such as water surface level and water surface slope. In particular, given that the chosen spatial resolution (every 10 km along river) matches the prospective resolution of the new Surface Water and Ocean Topography (SWOT) satellite this dataset can benefit the study of SWOT measurements over this system. The short time period of measurements is sufficient for feasibility and measurement evaluation studies related to SWOT.
• The concurrent discharge and water level data at high spatial and temporal resolution may be used to calibrate and validate both hydraulic and hydrological models and provide new insights on macro- and micro-pollutant transport, saline intrusion and urban flood dynamics.
• Even though provided for only two months, the resolution of this data enables a better scientific understanding of tidal wave propagation along the Saigon-Dong Nai estuary system by providing a high spatial resolution of measurements.

## 1 Objective

The dataset presented was collected during a 2-month field campaign in Ho Chi Minh City (HCMC), Vietnam within the context of a French-Vietnamese collaboration between the Centre Asiatique de Recherche sur L'Eau (CARE) and the Institut des Géosciences de l'Environnement (IGE). This particular campaign was carried out in connection with a hydraulic study to assess the new capabilities of the Surface Water and Ocean Topography (SWOT) satellite applied to the Saigon-Dong Nai estuary system. It includes water level and temperature measurements at five locations along the Saigon river and 2 locations along the Dong Nai river as well as discharge measurements from four 24-hour ADCP campaigns at 2 locations in the Saigon river and 1 location in the Dong Nai river. Additionally, water level was barometrically compensated using air pressure measurements. Data were sampled between October 21^st^, 2022 and December 16^th^, 2022 and are provided in raw (e.g. *.hobo* files), pre-processed (e.g. barometric compensation) and post-processed (e.g. quadratic interpolation) form [1]. A brief description of each data format is given below.

## 2 Data Description

The location of sensors and ADCP campaigns are presented in Table 1 together with their respective variables. The water level datasets are provided in files named as follows: “[Location ID]-[type].csv” or “[Location ID]-[type].hobo”. Similarly, the discharge datasets are provided in files named as follows: “[type]-[Location ID]-[date].csv”. The File IDs are summarized in Table 1 and the types refer to the level of processing namely, “raw”, “pre-processed” or “post-processed”. Details on the level of processing are given in sections 3.1 and 3.2.

**Table 1:** Summary of information on location of measurements, file ID and corresponding variables.

Location ID	River Basin	Lat. [°N]	Lon. [°E]	Type	Variables
Cu-Chi	Saigon	11.054518	106.539539	raw	Absolute pressure, temperature
				pre	Absolute pressure, temperature, Water level
				post	Corrected water level
				air	Absolute and relative air pressure
HOBO1	Saigon	10.931750	106.652340	raw	Absolute pressure, temperature
				pre	Absolute pressure, temperature, Water level
				post	Corrected water level
				ADCP	Discharge, discharge uncertainty and estimated error, width, cross-section
HOBO2	Saigon	10.869837	106.699607	raw	Absolute pressure, temperature
				pre	Absolute pressure, temperature, Water level
				post	Corrected water level
HOBO3	Saigon	10.829748	106.709833	raw	Absolute pressure, temperature
				pre	Absolute pressure, temperature, Water level
				post	Corrected water level
				ADCP	Discharge, discharge uncertainty and estimated error, width, cross-section
La garden	Saigon	10.785773	106.721540	raw	Absolute pressure, temperature
				pre	Absolute pressure, temperature, Water level
				post	Corrected water level
HOBO DN1	Dong Nai	10.944861	106.816343	raw	Absolute pressure, temperature
				pre	Absolute pressure, temperature, Water level
				post	Corrected water level
				ADCP	Discharge, discharge uncertainty and estimated error, width, cross-section
HOBO DN2	Dong Nai	10.678755	106.777768	raw	Absolute pressure, temperature
				pre	Absolute pressure, temperature, Water level
				post	Corrected water level

### 2.1 Water Level

*Raw files* – Files in *.hobo* format directly provided by each sensor. These files provide absolute pressure and temperature readings.

*Pre-processed files* – Raw files processed using HOBOware Pro v3.7.21 software in *.csv* format. These files contain water level compensated for barometric pressure changes.

**Fig. 1:**
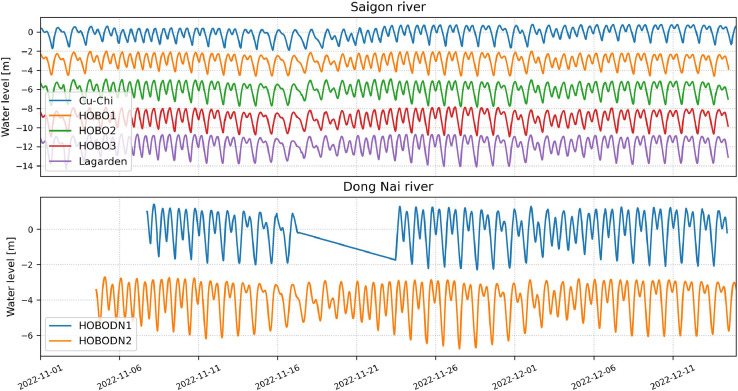
Post-processed water level measurements for the Saigon river (a) and for the Dong Nai river (b). For the sake of readibility, the water levels are discplaced by 3 meters in a) and by 4 meters in b). The HOBODN1 location shows a data gap due to a stolen sensor. This data corresponds to the post-processed water level data found in “[Location ID]-post.csv” files.

*Post-processed files* – Pre-processed files processed using Python in *.csv* format. These files contain water level data which was corrected for out-of-water periods, homogenized for reference point and normalized across sensors (Fig. 1).

### 2.2 Discharge

**Fig. 2:**
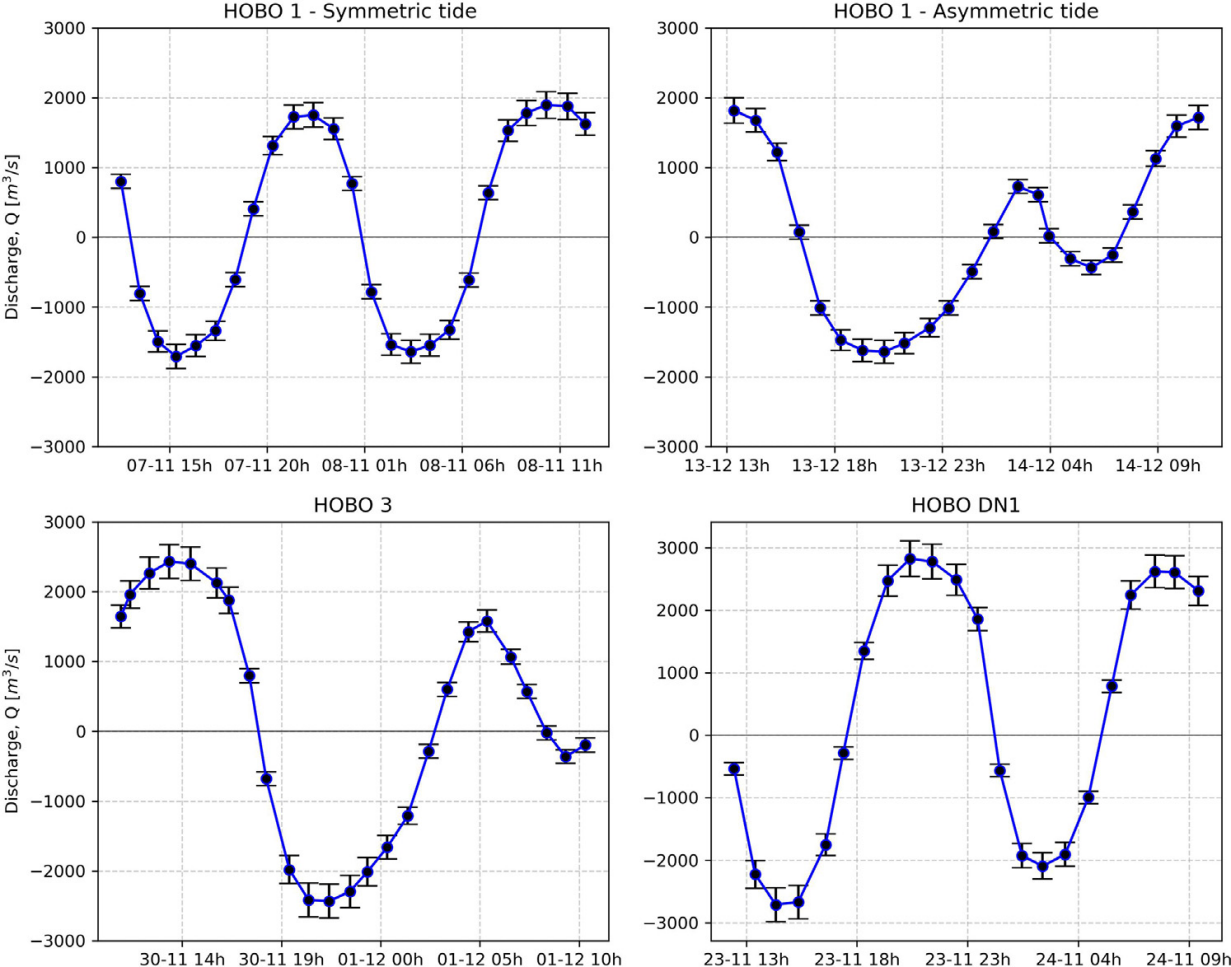
Plot of the post-processed ADCP measurements and corresponding error uncertainties. This data corresponds to the post-processed water level data found in “[Location ID]-ADCP.csv” files.

*ADCP files* – Files produced with QrevInt software and the OURSIN framework [2] in .csv format. These files contain mean discharge, mean width and mean river cross-section as well as the OURSIN discharge uncertainty and error estimation in the discharge measurement (Fig. 2).

## 3 Experimental Design, Materials and Methods

### 3.1 Water Level

The water level dataset comprises data for the locations summarized in Table 1 and was obtained using HOBO U20L-01 water level loggers at a time resolution of 15 minutes. This device has a typical error of ±0.1 %, a maximum error of ±0.2 % and a resolution of < 0.21 cm. The loggers were deployed using a low-cost brick-and-pipe system where the logger is secured inside a PVC pipe and attached to a brick. In order to minimize sediment deposition over the logger several holes are made on the PVC pipe and the pipe and brick are installed in such a way that the sensor is at least 15 cm off the bottom of the river. Prior to the first deployment all loggers were tested and calibrated using a water column.

#### 3.1.1 Pre-Processing

Pre-processed data includes the HOBO logger records of absolute pressure, which are converted to water level readings by HOBOware Pro software version 3.7.21. In this application, absolute pressure includes atmospheric pressure and water head. Atmospheric pressure is nominally 100 kPa at sea level, but it changes with weather and altitude. To compensate for barometric pressure changes, we use measurements from a Watson W-8681-MKII wireless weather station as barometric reference. This reference was deployed at the Cu-Chi location. Barometric pressure readings are consistent across a region (except during fast-moving weather events), thus it is generally correct to use barometric pressure readings that are taken within 30 km of the logger without significantly degrading the accuracy of the compensation. Therefore, this weather station was used to compensate all the water level loggers in the area. The HOBOware Pro software uses the hydrostatic equation to calculate water level from pressure measurements. The level of water can be calculated from the hydrostatic pressure using the formula:

(1) $$h=\frac{P}{\rho \cdot g}$$

where *h* is the height of the liquid column [m], *P* is the pressure, *ρ* is the density of the liquid [kg/m³], and *g* is the gravitational acceleration [m/s²]. However, the column of air above the water will affect the pressure readings due to fluctuations in atmospheric pressure. The measured pressure at the bottom of the column of water will be the sum of the hydrostatic pressure due to the weight of the water and the atmospheric pressure due to the weight of the air above it. To accurately measure the water level, the atmospheric pressure must be subtracted from the pressure readings. This computation is performed using the HOBOware Pro dedicated tool and is refered to as barometric compensation.

#### 3.1.2 Post-Processing

Post-processed data includes water level that has been corrected for out-of-water periods, homogenized for reference point differences and normalized across sensors.

The temperature signal was used to identify out-of-water periods. During these periods the sensor captures the air temperature rather than the water temperature creating temperature anomalies. We remove water level readings below the 2^nd^ and above the 98^th^ percentile of temperature over the measurement period. We fill these values by using quadratic interpolation [3] and implement it using the interpolate function in Python. Quadratic interpolation is a numerical method used to estimate missing values in a dataset. It involves fitting a quadratic function, which is a second-order polynomial of the form

(2) $$y=ax^{2}+bx+c$$

to the data points surrounding the missing values. The coefficients *a, b*, and *c* are determined by solving a system of equations using the known data points. Once the quadratic function has been fitted to the data, it can be used to estimate the values of the missing data points by evaluating the function at their x values. This provides a smooth curve that passes through all of the known data points and can be used to estimate the values of the missing data points.

In order to retrieve the data, the logger needs to be removed and re-placed in the water. This means that the position of the sensor slightly changes every time data is retrieved. Hence, the reference point changes between data retrievals. In order to mitigate this effect, the following methodology was applied. Given a water level signal *y* and a retrieval of data *m* times during the campaign, for each time period between data retrievals, the average water level, *a*_*i*_, can be computed for the *i*^*th*^ time period. Then, we substract the average water level a_i_ from the signal *y*_*i*_ in that time period to obtain the corrected signal *y*_*corrected*_:

(3) $$y_{corrected_{i}}=y_{i}-a_{i}$$

This process can be repeated *m* times for each time period between data retrievals to obtain the corrected water level signal over the full time period of measurements. This helps to improve the accuracy and consistency of the water level data.

At each sensor location, the reference point is different and unknown. In order to be able to compare the signals at different locations we normalize all signals. One way to do this is by using a method called mean normalization. This involves calculating the mean water level for each sensor over the full time period of measurements and then subtracting this mean from the water level signal for that sensor. This is done for each sensor using Eqn 3 applied to the full period of measurements.

### 3.2 Discharge

The discharge dataset comprises data for the locations summarized in Table 1 and was acquired using the Teledyne Workhorse Rio Grande Acoustic Doppler Current Profiler (ADCP). Four 24-hour measurement campaigns were performed. Two transects were made every hour yielding one discharge measurement per hour.

#### 3.2.1 Post-Processed Files

Post-processed ADCP discharge files include mean discharge, mean width and mean river cross-section. Additionally, these files include discharge uncertainty calculated using the OURSIN framework [2] and error estimation in the discharge measurement.

The mean discharge, mean width and mean river cross-section are taken over two transects every hour. The discharge uncertainty is calculated using the OURSIN method. The OURSIN method is a framework for computing the uncertainty of moving-boat ADCP discharge measurements that has been implemented in QrevInt software. The uncertainty of extrapolated discharges in unmeasured areas is estimated by varying the maximum number of parameters, as an alternative to the Monte Carlo approach. A uniform distribution of errors is assumed so that any value in the minimum-maximum possible range is equally likely to occur. The OURSIN method also accounts for systematic versus random errors in the computation of the uncertainty of discharge measurements averaged over repeated ADCP transects. Then, the decomposition of the different error sources allows determining their influence on the overall uncertainty [4]. In addition, the error in ADCP measurements was evaluated at 10% using a minimum error value of 100 m^3^/s given the adverse conditions encountered during the campaigns [5,6].

## CRediT Author Statement

**Francisco Rodrigues do Amaral:** Conceptualization Methodology, Investigation, Data curation, Writing, Review & Editing; **Tin Nguyen Trung:** Methodology, Data curation; **Nicolas Gratiot:** Supervision, Review & Editing; **Thierry Pellarin:** Supervision, Review & Editing.

## Declaration of Competing Interest

The authors declare that they have no known competing financial interests or personal relationships that could have appeared to influence the work reported in this paper.

## Data Availability

High-resolution water level and discharge measurements from the Saigon-Dong Nai estuary system, Vietnam, 2022 (Original data) (Dataverse).
